# Biohybrid microvascular interponates with integrated elastin-like recombinamers – validation of stability and biomimetic elasticity in human vessels

**DOI:** 10.1038/s41598-025-33635-x

**Published:** 2025-12-25

**Authors:** Marius Heitzer, Dominic Andre, Philipp Winnand, Mark Ooms, Nils Vohl, José Carlos Rodríguez-Cabello, Stefan Jockenhoevel, Frank Hölzle, Ali Modabber, Alicia Fernández-Colino

**Affiliations:** 1 Department of Oral and Cranio-Maxillofacial Surgery, University Hospital RWTH Aachen, Pauwelsstraße 30, 52074 Aachen, Germany; 2 Department of Biohybrid & Medical Textiles (BioTex), AME-Institute of Applied Medical Engineering, Helmholtz Institute, RWTH Aachen University, Forckenbeckstrasse 55, 52074 Aachen, Germany; 3 BIOFORGE Lab, University of Valladolid, CIBER-BBN, Paseo de Belén 19, LaDIS, Valladolid, 47011 Spain

**Keywords:** Vascular grafts, Elastin-like recombinamers, Biohybrid, Microvessels, Maxillofacial surgery, Biotechnology, Materials science, Medical research

## Abstract

Autologous vein grafts remain the gold standard for microvascular interpositions in reconstructive maxillofacial surgery, despite their high failure rates and the additional operation site. Against this background, this ex vivo study investigates a novel biohybrid microvascular graft based on elastin-like recombinamers (ELRs). The biohybrid graft design consists of ELRs combined with the mechanical support of textile components. The compliance and pressure resistance of the developed grafts were evaluated and compared with human fresh cadaver vessels (radial artery). The ELRs biohybrid grafts were microsurgically anastomosed with human radial arteries, and were compared with human vein interposition grafts from the saphena vein. The ELRs grafts exhibited comparable and favorable compliance to real human vessels. The burst pressure values of 543 ± 71 mmHg of the ELRs grafts were above the physiological blood pressure load. The tensile strength of the interponated ELRs grafts were significantly higher at 4.2 ± 1.15 N (*p* = 0.025) compared to interponated venous grafts with tensile strength of 3.20 ± 0.65 N. The ex vivo investigations of the novel ELRs biohybrid microvascular grafts revealed promising properties for the application as microvascular interposition graft in terms of compliance, pressure resistance, and tensile strength compared to the current gold standard.

**Supplementary Information:**

The online version contains supplementary material available at 10.1038/s41598-025-33635-x.

## Introduction

Complex tissue and bone defects in the oral, and maxillofacial regions, as well as in many other medical fields, often require for reconstruction the transplantation of free microvascular flaps to improve functional and aesthetic outcomes^1,2^.

A free microvascular flap is a surgical technique used in reconstructive surgery where a section of tissue—such as skin, muscle, bone, or a combination—is completely detached from its original location (the donor site) along with its supplying blood vessels. This tissue is then transplanted to a different area of the body (the recipient site) where reconstruction is needed. The key feature of a free microvascular flap is that the blood vessels supplying the flap are reconnected (anastomosed) to blood vessels at the recipient site using microsurgical techniques. This allows the transplanted tissue to survive and function in its new location. An adequate vessel length is the fundamental prerequisite for a tension-free and reliable microvascular anastomosis, thereby reducing the risk of anastomotic failure, which can have devastating effects on free microvascular flap perfusion and can lead to its failure^2,3^. However, damaged recipient vessels, defect location, trauma sequelae, atherosclerosis, or previous radiation therapy present significant limitations for the use of free microvascular grafts and frequently result in inadequate vascular pedicle lengths^2,4^. Consequently, the choice of the free vascular flap must currently be based primarily on the length of the vascular pedicle, even if the chosen flap may be inferior to other microvascular flaps in terms of aesthetics and functionality in the recipient area.

To broaden the range of suitable free flaps for optimal tissue reconstruction, the vascular pedicle often needs to be lengthened to ensure it reaches the recipient vessels^2^. The current gold standard for vascular pedicle extension is the use of autologous interposition grafts, in which a venous segment from another vessel of the patient is utilized^5,6^. However, harvesting an autologous interposition graft often requires an additional surgical site, leading to further comorbidities such as wound infections, postoperative pain, and bleeding^7^. Moreover, the use of autologous vascular interposition grafts in microvascular transplantation is associated with significant complication rates of 17.1% and graft loss rates of 11%, as reported in the literature^2^. Due to these high complication rates of the gold standard, there is a substantial need for alternative approaches and new methods.

In other medical fields, the use of vascular interposition grafts for larger arterial vessels, such as coronary bypass surgery, is a well-documented and extensively researched approach^8^. In addition to autologous interposition grafts, alloplastic vascular interposition grafts made of polyethylene terephthalate or polytetrafluoroethylene are established therapeutic options^7,8^. However, these alloplastic materials are subject to considerable limitations, particularly as small vascular interposition grafts with a diameter of < 6 mm. These limitations include rheological phenomena, such as a significantly increased risk of thrombosis due to a striking compliance mismatch between the vessel wall of the artery and the vascular interposition graft^9^, pronounced intimal hyperplasia^10^, and insufficient long-term perfusion^7,11,12^.

In contrast to large arterial bypasses, the significantly smaller diameter of the vascular pedicles of microvascular grafts—ranging from diameters of 1.4 mm to 1.6 mm^13^—has so far posed considerable constraints on the clinical use of alloplastic vascular interposition grafts. Rigid and small alloplastic vascular interposition grafts with a diameter of 1 mm have already been studied in the literature in small animal models^14^.Due to their lack of elastic properties and a high mismatch of vessel wall compliance, these interposition grafts exhibited failure rates of 78.4% ^14^. Existing technical challenges in biomaterial science include mimicking the design of natural systems. In particular, mimicking the natural extracellular matrix remains a demanding and important challenge^15^. Especially when manufacturing small vascular prostheses with a diameter of 1 mm, it is important to achieve elastic and anisotropic properties that correspond to the natural tissue properties and the vessel wall compliance of small blood vessels.

Elastin represents a major component of the extracellular matrix and plays a crucial role in natural tissues, significantly influencing their mechanical properties and biological functions^16^. Its absence in medical implants, particularly vascular grafts, can lead to severe complications such as stenosis and thrombosis, ultimately resulting in graft failure. The flexible and elastic properties of natural elastin are associated, among other factors, with the hydrophobic pentapeptide Val-Pro-Gly-Val-Gly (VPGVG)^17^. Elastin-like recombinamers (ELRs) represent a family of artificial polymers derived from VPGVG and variations thereof^17,18^. In addition to their elastic mechanical properties, recombinant ELRs have been reported in the literature to exhibit excellent hemocompatibility and favorable bioactivity^9,17,19,20^. Moreover, in vitro studies have demonstrated that ELRs provide a non-thrombogenic surface^21^, favor cell ingrowth, and enables the development of an endothelial cell layer^9,19,20^.

Therefore, numerous studies have identified elastin and elastin-like polymers as promising candidates for the development of advanced blood-contacting devices^22–24^. The valuable elastic mechanical properties (i.e. reversible contraction and expansion) can significantly improve the compliance of alloplastic vascular interposition grafts and thus their functionality. By precisely controlling the composition of the ELRs selected for the graft manufacturing, further properties can be finely tuned to meet specific requirements, like degradation rate and specific cellular adhesion among others^18^. Therefore, the incorporation of elastin-based components, such as ELRs, into alloplastic vascular grafts has the enormous potential to significantly improve the in vivo performance of alloplastic vascular prostheses.

We have previously described larger vascular grafts^9^, venous valves^19^, and heart valves^25^ made of ELRs, which have demonstrated adequate hydrodynamic performance, favorable cellular responses as well as structural resilience during ex vivo experiments. Building on these promising findings, this ex vivo study with human vessels aims to investigate a novel miniaturized biohybrid vascular graft. The objective is to combine the elastic and bioactive properties of ELRs with the mechanical stability of textile components to overcome the limitations and high failure rates associated with autologous or previously described rigid microvascular interposition grafts.

## Materials and methods

### Fabrication of biohybrid ELR-microvascular graft

The biohybrid microvascular grafts were produced in the laboratory of the Department of Biohybrid & Medical Textiles (RWTH - Aachen University, Forckenbeckstrasse 55, 52074, Aachen, Germany). For the matrix material of the alloplastic vascular grafts, the structural ELR, named as VKV (see Table S1 for complete sequence) were used. Lysine residues were chemically modified as previously reported^26^ to introduce cyclooctyne and azide groups. The resulting derivatized variants, e.g. VKV-cyclooctyne and VKV-azide, were crosslink via catalyst-free click chemistry to form a hydrogel.

The ELR hydrogel was reinforced with a warp-knitted polyethylene terephthalate (PET) textile. For that, each ELR component (100 mg mL⁻¹) was dissolved for 1 h in a 1:1 (v/v) mixture of PBS (pH 7.4, Gibco, Life Technologies, Carlsbad, CA, USA) and ethanol (Supelco, EMSURE^®^, Sigma-Aldrich, St. Louis, MO, USA). The solutions were sequentially loaded into the same syringe, mixed by inverting the syringe 20 times, and injected into the mold.

The mold consisted of a custom-made polycarbonate (PC) outer shell (inner diameter = 2 mm) and a polished stainless steel inner core cylinder (outer diameter = 1 mm) arranged coaxially. The components were assembled and sealed by screwing them into a polyoxymethylene base before clamping the two shell halves together. The heat-set PET textile was positioned concentrically in the annular space using spacers at both ends. The PET mesh (1 mm diameter tricot, 1 × 1 lapping, EAC 14) was produced in-house on a double-needle bar Raschel machine (MiniTronic 800, Rius-Comatex, Barcelona, Spain). It was draped over a stainless-steel hollow core (outer diameter = 1.5 mm), secured with clamps, and heat-set by autoclaving at 121 °C for 20 min.

During molding, the ELR mixture was injected from the bottom. Crosslinking occurred at room temperature for 30 min, after which the shell was opened and the ELR-textile scaffold carefully removed from the core. The graft was then placed in 70% (v/v) ethanol overnight, washed in sterile PBS (3 × 30 min, followed by 1 × 1.5 h at room temperature), and autoclaved at 121 °C for 15 min. (dx-serie, Systec, Germany)^27^, submerged in 60 mL PBS and stored in the autoclaving container until characterization.

### Human vascular grafts

All procedures involving human tissues were performed in accordance with the 1964 Declaration of Helsinki and its later amendments or comparable ethical standards. The experiments involving human tissues were conducted in accordance with the ethical approval given by the Ethics Committee of the Medical Faculty of RWTH Aachen, Germany (EK 219/16) and from the Institute of Molecular and Cellular Anatomy at the University Hospital RWTH Aachen. The donors on which this study was based provided written informed consent and permission during their lifetime for their bodies to be used after death for research and education purposes. In total, 15 vascular pedicles of the radial artery were harvested from 8 human fresh-frozen cadavers (3 female and 5 male; average age 75.6 years, range 65–86 years). Moreover, to compare the alloplastic interposition grafts with the gold standard, 5 small saphenous veins were harvested. Exclusion criteria for the use of human vessels were clinical signs of atherosclerosis, disrupted integrity of vessel walls, diameter > 1.4 mm and existing thrombusformation within the lumen. All surgical procedures were performed by a highly experienced person with several years of practical experience with microsurgical anastomoses. The fine dissection of the vessels from the surrounding connective tissue was performed on a dissection table using a surgical microscope (Carl Zeiss Meditec AG, Jena, Germany), and the tissue was continuously kept in phosphate-buffered saline (PBS) solution to prevent dehydration. (Gibco, pH: 7.4, Life Technologies, Carlsbad, CA, USA). The distal part of the arteria radialis was dissected from the two commitant veins. Side branches of the radial arteries were clipped with Weck microvascular clips (Teleflex, Wayne, PA, USA). For the compliance tests five of the radial arteries were dissected into 20 mm long sections.For tensile testing and burst pressure testing, the other ten arteries were cut in the middle into 10 mm long specimen sections. The interposition specimen of the small saphenous veins and the alloplastic biohybrid interponated grafts had a length of 10 mm and were anastomosed by single sutures with Ethilon 9 − 0 (Ethicon, Livingston, United Kingdom) in between the two 10 mm long arterial specimen sections. To ensure standardized conditions for testing, each anastomosis was created using 9 single-button sutures with the same stitch spacing. In total five artery-vein-artery (AVeA) interpositions and five artery-graft-artery (AGrA) interpositions were created. The specimens with the interposed saphenous vein served as control.

### Vessel compliance test

Five radial arteries with a length of 20 mm and five biohybrid microvascular grafts with ELRs with same length of 20 mm (biohybrid graf, inner diameter = 1 mm) were mounted in a custom-made bioreactor system as previously described^9^. The bioreactor chamber is made of polyoxymethylene (POM; Licharz GmbH, Buchholz, Germany) with clear poly(methyl methacrylate) (PMMA) sides. An optical micrometer model LS-7030(M) (Keyence Deutschland GmbH, Neu-Isenburg, Germany) was used to measure the outer diameter of the artery/biohybrid microvascular graft by registering the size of the shadow on the sensor positioned across a parallel green laser from the transmitter. The system additionally featured silicone tubes (Ismatec™), an adjustable resistance to control the flow and pressure within the system, and two in flow pressure sensors, one before and after the chamber (Xtrans, Codan pvb Medical GmbH, Germany). The loop was powered by a small centrifugal pump (702–6882, RS components, Corby, UK), which was controlled by a customized control unit^28^. PBS heated at 37 °C, was used to fill the flow loop. The mean pressure was adjusted through modulating the pulse-free flow by changing the voltage delivered to the centrifugal pump, in combination with the adjustable resistance. The pressure pulses (20/60, 30/70, 40/80, 50/90, 60/100, 80/120, 110/150 mmHg) were created, by changing the constant input voltage, using a sinusoidal function. The applied pressures and corresponding diameters were recorded with a custom-developed LabVIEW program (LabVIEW 20.1, National Instruments). The average values of the compliance were calculated using the following formula:

$$\:C=\frac{(\frac{{D}_{2}{-D}_{1}}{{D}_{1}}\times\:{10}^{4})}{{P}_{2}{-P}_{1}}\:\:\left[\frac{\%}{100\:mmHg}\right]$$

P1 is the lowest internal pressure, P2 is the highest internal pressure, D1 is the diameter at the pressure P1 and the D2 is the diameter at the pressure P2. The compliance of each graft was calculated from the meaning of the compliance values extracted from the recorded cycles. *n* = 5 samples were tested for each group (ca. 60 cycles per pressure range).

### Burst pressure test

For the evaluation of burst strength, we tested tubular samples, in accordance with the ISO 7198 requirements. The samples (*n* = 5, length = 20 mm) were mounted on a custom-made burst pressure chamber equipped with a pressure sensor (Jumo Midas pressure transmitter; JUMO GmbH & Co. KG, Fulda, Germany) and a NE-1000 SyringeONE Programmable Syringe Pump (New Era Pump Systems, Inc; Farmingdale, NY, USA)^9^. The samples were completely filled with PBS (Gibco, pH:7.4, Life Technologies, Carlsbad, CA, USA), closed using a stopper, and immersed in the PBS buffer at 37 °C for 10 min before the measurement started. The pressure in the system was increased by pumping PBS at a constant rate of 20 mL/min until the structural failure of the sample was detected as a sudden drop in the pressure recorded by LabVIEW (National Instruments). The highest measured pressure value before failure was defined as the burst strength value and was recorded in mmHg.

### Tensile strength test

The tensile strength was measured using a UniVert uniaxial tensile tester (CellScale Biomaterials Testing, Waterloo, Canada) equipped with a 10 N load cell. The samples consisted of either a human small saphenous vein interposed between two human radial arterial segments (AVeA) or the ELRs biohybrid microvascular grafts (AGrA) also interposed between two human radial arterial segments. To maintain the tubular shape of the samples, their ends were inserted into rods and secured within the clamps of the tensile tester at both ends. All tests were performed with the samples fully immersed in PBS (Gibco, pH 7.4, Life Technologies, Carlsbad, CA, USA) at 37 °C. Tensile force was applied by moving one clamp away from the stationary clamp bearing the load cell at a rate of 50 mm/min, following ISO 7198 guidelines. The force at failure was recorded in newton (N). For each group five measurements were performed.

### Statistical analysis

All analyses were conducted using GraphPad Prism 10.1.1 (GraphPad Software, Inc., La Jolla, CA, USA). The Shapiro-Wilk test was used to test normal distributions. All data were tested as not normally distributed and nonparametric data were analyzed using the Mann-Whitney U test. Results are presented as mean ± standard deviation (SD). Statistical significance was defined as *p* ≤ 0.05.

## Results

### Morphological characterization of the biohybrid ELR microvascular grafts

**Fig. 1:**
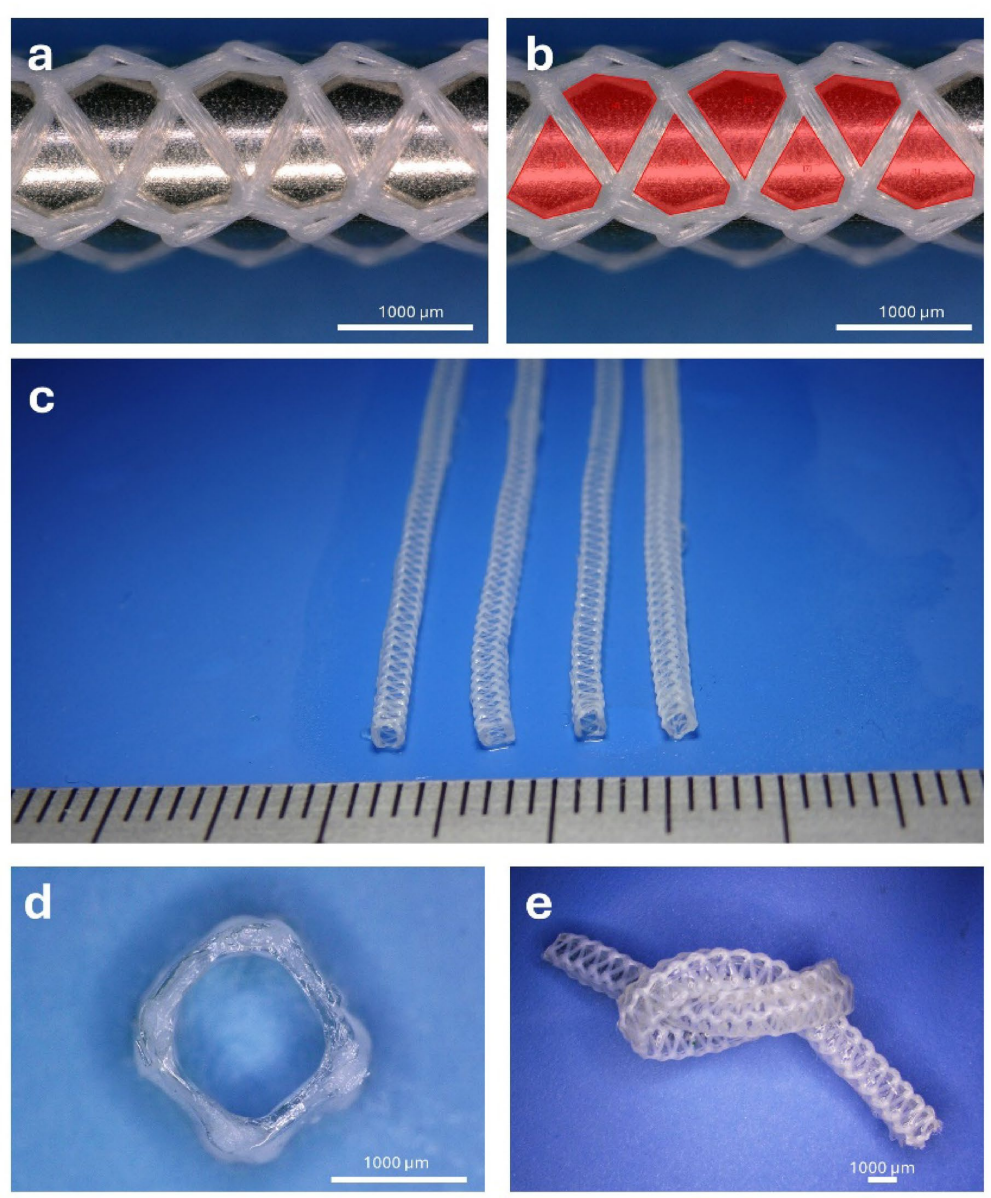
Morphological characterization of the biohybrid ELR microvascular grafts. (**a**) PET textile used as a core reinforcement of the graft. (**b**) PET textile with pores pseudo-colored in red to facilitate dimensional evaluation. (**c**) Side-by-side comparison of four identical grafts, composed of the PET textile coated with the ELR. (**d**) detailed view of the graft cross-section (lumen). (**e**) Image showcasing the flexibility of the developed microvascular grafts, that can be bent and knotted without kinking.

The developed microvascular grafts featured a PET textile (Fig. 1a) serving as the core structural component, which was integrated into an ELR matrix. The PET-textile pore size after heat setting was 0.284 ± 0.035 mm^2^. The grafts showed a homogenous appearance (Fig. 1c). A detailed examination of the graft cross-section revealed the smooth, well-defined lumen structure, confirming the integrity of the composite design (Fig. 1d). The grafts possessed an inner diameter of 1199 ± 19 μm and a wall thickness of 186 ± 41 μm. Finally, the flexibility of the fabricated grafts was demonstrated, as they could be bent and knotted without exhibiting any signs of kinking (Fig. 1d), highlighting their excellent mechanical adaptability.

### Compliance of human arteries and biohybrid microvascular grafts

**Fig. 2:**
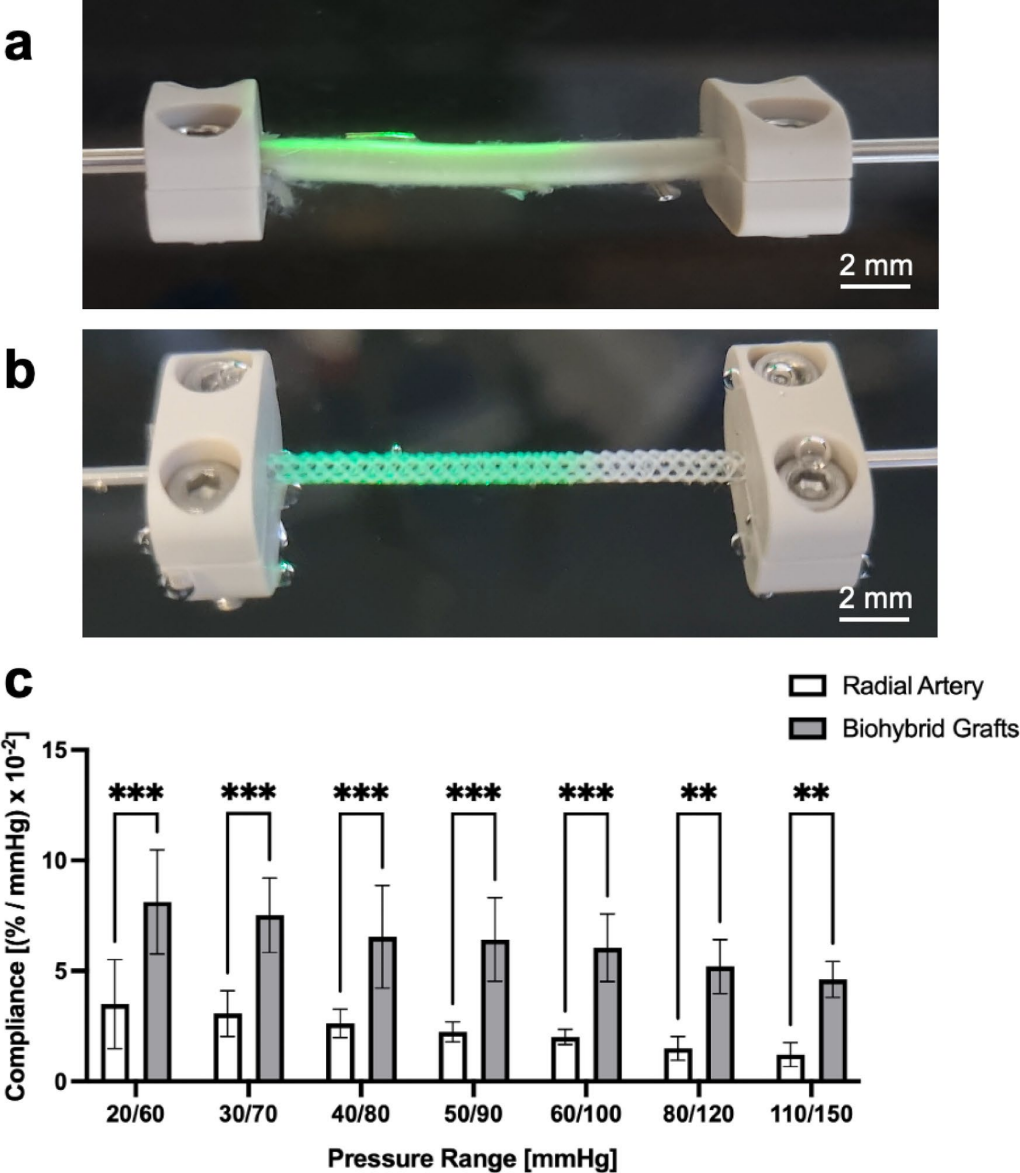
Compliance testing in the custom-made bioreactor system: (**a**) Picture of a clamped radial human artery. (**b**) Image of a clamped microvascular biohybrid graft made of ELRs. (**c**) Graphical representation of compliance testing of radial artery and biohybrid microvascular graft. ***: *p* < 0.001; **: *p* = 0.001.

The human arteries exhibited a clear trend of decreasing compliance with increasing pressure (Fig. 2). Specifically, the compliance values were 3.5 ± 1.8% mmHg⁻¹×10⁻² (20/60), 3.1 ± 0.9% mmHg⁻¹×10⁻² (30/70), 2.6 ± 0.6% mmHg⁻¹×10⁻² (40/80), 2.2 ± 0.4% mmHg⁻¹×10⁻² (50/90), 2.0 ± 0.3% mmHg⁻¹×10⁻² (60/100), 1.5 ± 0.5% mmHg⁻¹×10⁻² (80/120), and 1.2 ± 0.5% mmHg⁻¹×10⁻² (110/150). Our biohybrid microvascular grafts demonstrated significantly higher compliance than the native counterparts across all pressure ranges, with values of 8.1 ± 2.0% mmHg⁻¹×10⁻² (20/60), 7.5 ± 1.5% mmHg⁻¹×10⁻² (30/70), 6.5 ± 2.0% mmHg⁻¹×10⁻² (40/80), 6.4 ± 1.6% mmHg⁻¹×10⁻² (50/90), 6.0 ± 1.3% mmHg⁻¹×10⁻² (60/100), 5.2 ± 1.1% mmHg⁻¹×10⁻² (80/120), and 4.6 ± 0.7% mmHg⁻¹×10⁻² (110/150). Notably, while the ELRs biohybrid microvascular grafts exhibited higher absolute compliance, they followed a similar trend as native arteries, with greater compliance at lower pressure ranges and a progressive decline at higher pressures.

### Burst pressure test

**Fig. 3:**
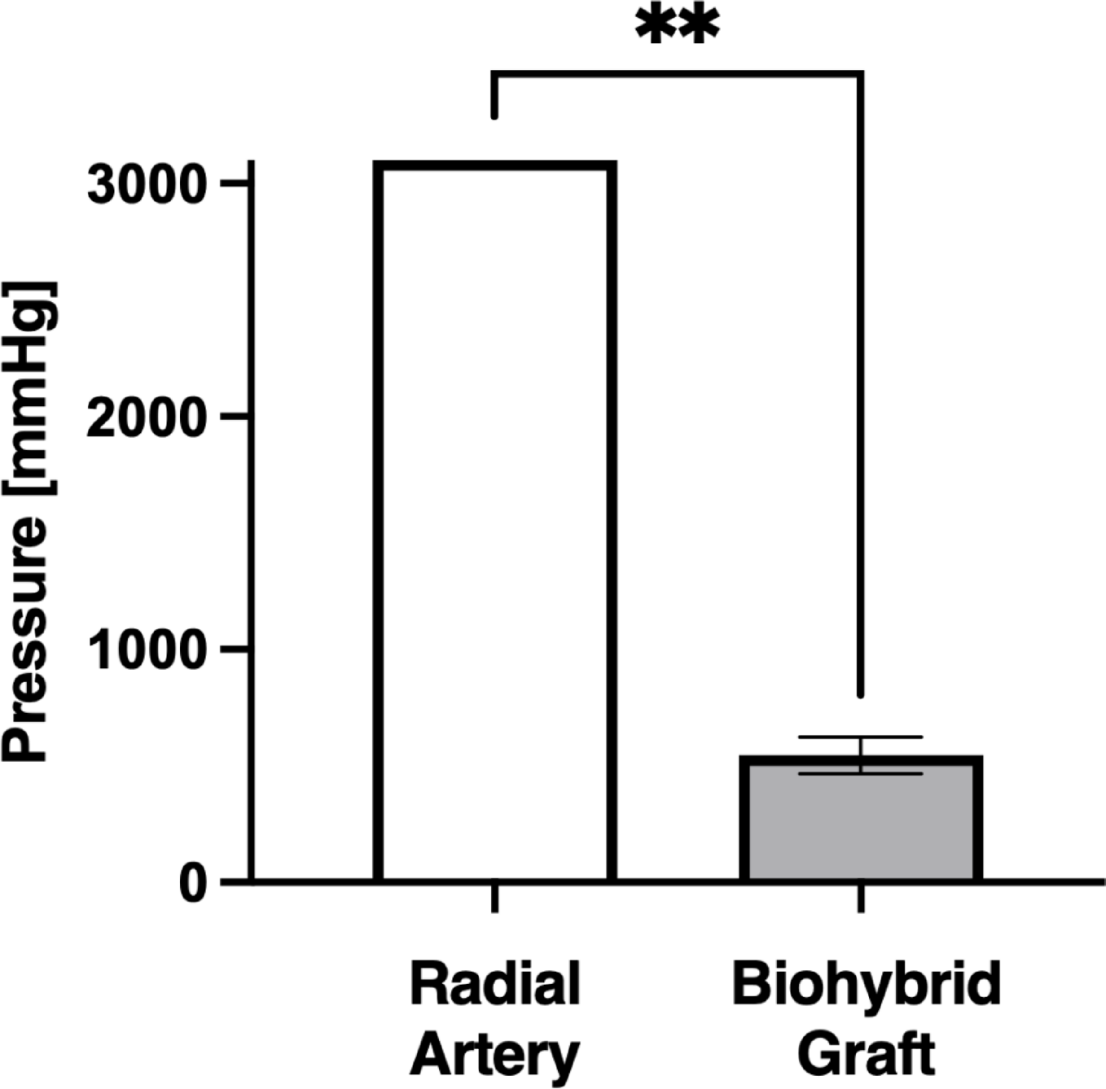
Graphical representation of burst pressure testing of radial artery and biohybrid microvascular graft. **: *p* = 0.008.

The ELRs biohybrid microvascular grafts had a burst pressure of 543 ± 71 mmHg (Fig. 3). Testing of the human radial arteries had to be terminated at 3000 mmHg due to limitations of the experimental setup, which was unable to withstand higher pressures. Consequently, the actual maximum burst strength of the human arteries exceeds the burst pressure value of the ELRs biohybrid microvascular grafts.

### Tensile strength of the ELR-graft anastomosed to human vessels

**Fig. 4:**
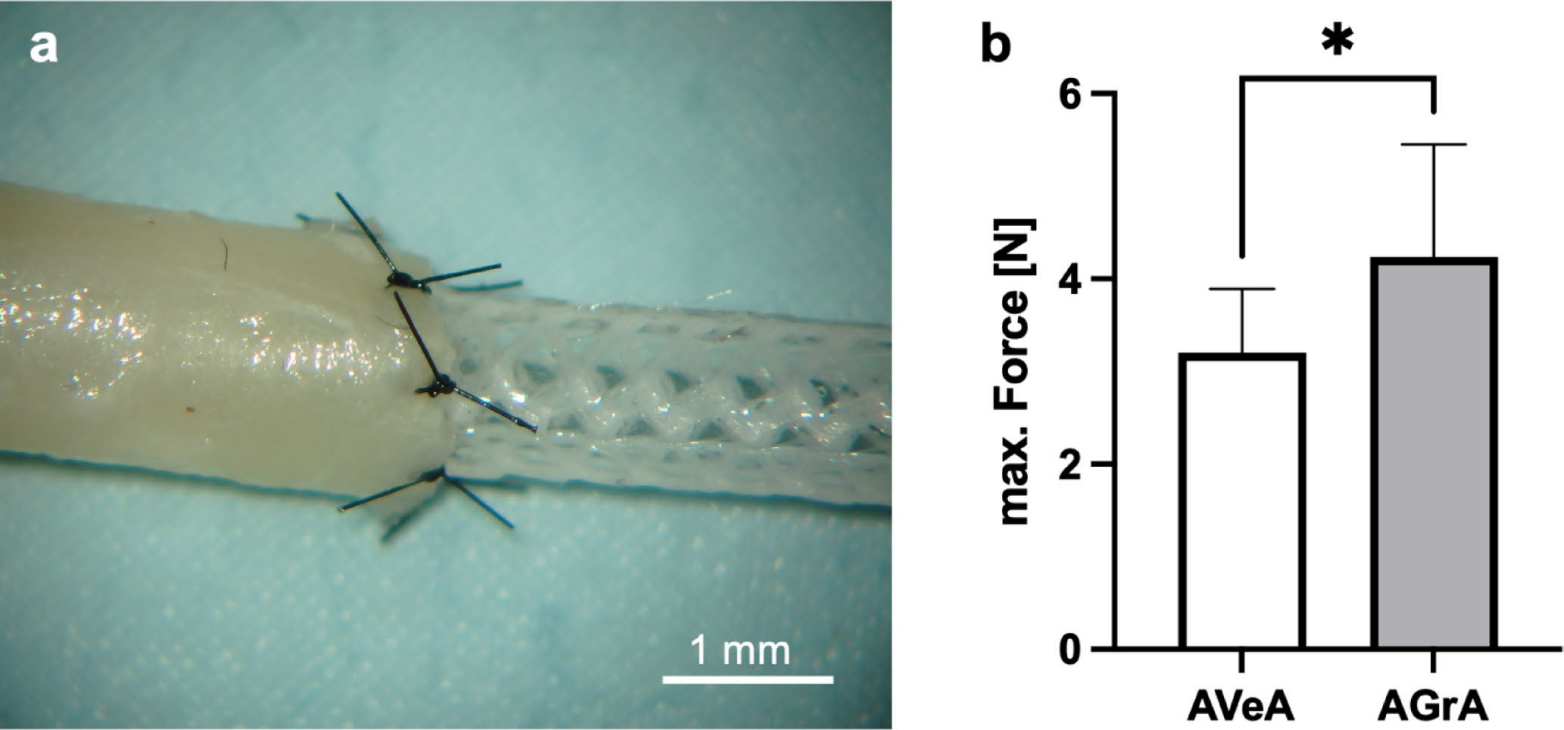
(**a**) Image of the microvascular anastomosis between human radial artery and biohybrid microvascular graft made of ELRs. (**b**) Graphical representation of tensile testing of radial artery with interponates of a human vein or the biohybrid microvascular graft. *: *p* = 0.025. Abbreviations: AVeA = Artery-Vein-Artery; AGrA = Artery-Graft-Artery.

All artery-vein-artery (AVeA, *n* = 5) and artery-graft-artery interpositions (AGrA, *n* = 5) were successfully performed, demonstrating the suitability of our grafts to be sutured and their ability to withstand surgical handling. The AGrA samples with the interposed ELRs biohybrid microvascular grafts exhibited a significant higher anastomotic strength of 4.2 ± 1.15 N (*p* = 0.025) than the AVeA counterparts. The AVeA samples with the vein interposition demonstrated a breaking strength of 3.20 ± 0.65 N (Fig. 4).

**Fig. 5:**
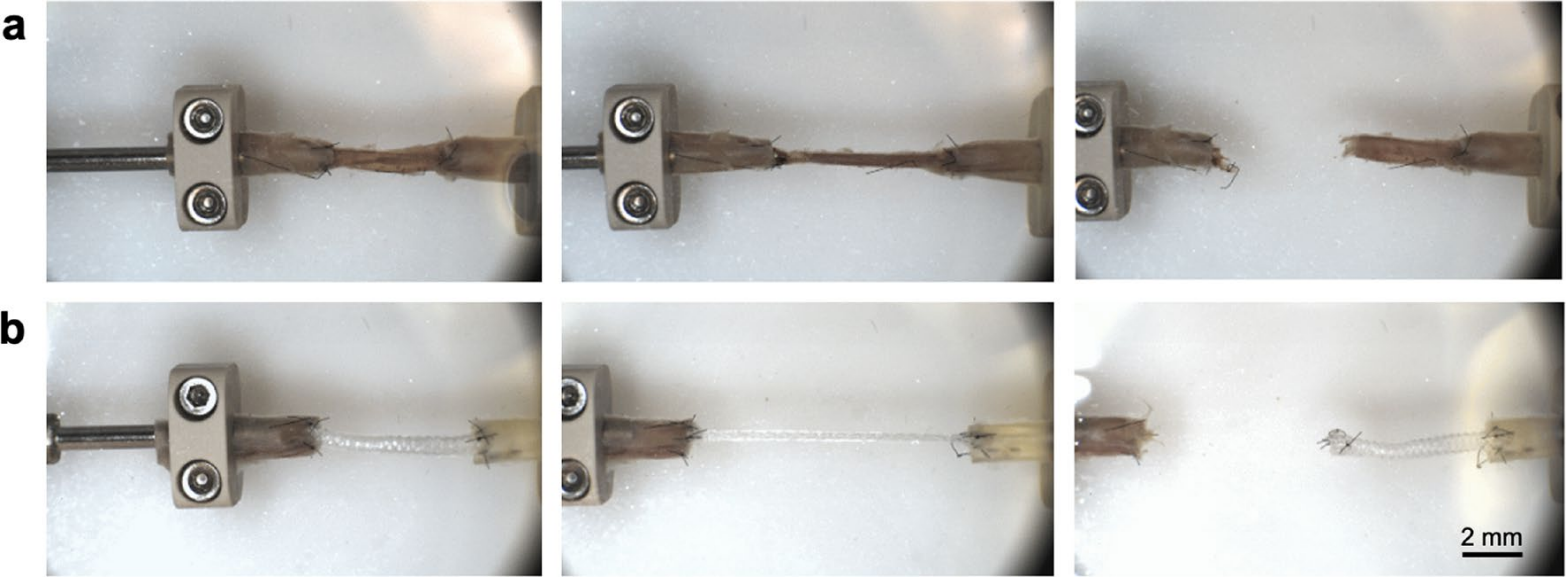
Images of tensile strength measuring using a UniVert uniaxial tensile tester. The force of the tensile test increases from left to right in the series of images. (**a**) Radial artery with interposition of vein graft (AVeA). The time points for the figures are as follows (from left to right): t1 = 0 s, t2 = 10 s, t3 = 15s. (**b**) Radial artery with interposition of biohybrid microvascular graft (AGrA).; The time points for the figures are as follows (from left to right): t1 = 0 s, t2 = 15 s, t3 = 20s.

The AVeA specimens failed during the tensile pulling due to vein rupture, while the AGrA specimens failed due to either rupture of the suture material or rupture of the vessel walls of the human arteries. The ruptures of the vessel walls occurred in the areas where the microsurgical suture material was inserted (Fig. 5).

## Discussion

In reconstructive surgery, the selection of a free microvascular flap for the treatment of a complex tissue defect is often based on the length of the flap’s pedicle. This means that a large number of potential microvascular flaps with better aesthetics and functionality cannot be used due to insufficient vessel pedicle length. Thus, extension procedures with vascular interponates not only expand the range of possible free flaps, but also enable the selection of the most suitable ones.

Autologous venous interposition grafts remain the gold standard for extending short vascular pedicles of microvascular flaps^5,6^, but suffer from additional donor site morbidity^7^ and increased complication rates^2^ that may lead to graft failure. Indeed, autologous vascular interposition grafts have been reported to have complication rates of 17.1%^2^. An undeniable factor contributing to the relatively high complication rates and poor survival rates of free flaps is the use of autologous vascular interposition vein grafts^29^, with reported overall failure rates of microvascular flaps of up to 11% of cases^2^. On the other hand, the inherent drawbacks of this technique, particularly regarding a potential association with flap compromise, must also be considered. Common causes for the high complication rates of venous interposition grafts are their potential thrombogenicity and their high susceptibility to pedicle kinking, twisting or stretching^30^. Furthermore, rheological factors, such as intravascular turbulences^31^, can contribute to the development of intravascular thrombosis of venous interposition grafts.

Various vascular interposition grafts have been presented in the past^7,9,11,12^, typically featuring dimensions bigger than 3 mm. Especially for alloplastic vessel grafts with a diameter of 1 mm, rigid vessel walls with insufficient elasticity result in insufficient compliance and ultimately in significant failure rates of 78.4%^14^.. Such compliance mismatch results in haemodynamic flow changes at the microvascular anastomosis of the alloplastic vascular interposition graft and this can lead to thrombotic obstruction of the vascular lumen and intimal hyperplasia, which is ultimately associated with reduced patency and increased failure of the graft^32,33^. Therefore, there is a clear medical need for alloplastic vessel grafts able to feature (i) an native-like elastic performance to strech and recoil, as well as (ii) the strenght to withstand the anastomosis procedure and the arterial blood pressure.

This study address for the first time the fabrication of biohybrid vascular grafts of particularly small dimensions (diameter 1 mm) and its microsurgical suitability to be anastomosed to human vessels. The design of the microvascular grafts presented in this study is based on a biohybrid concept. Specifically, a warp-knitted PET mesh was utilized as reinforcement to maintain the structural integrity and mechanical stability of the alloplastic graft, while an ELR matrix (bioinspired in the native elastin of human tissues) imparts flexibility and biomimetic elasticity to the artificial vessel.

One factor contributing to their higher complication rates of vein grafts is their inferior compliance relative to that of human arterial vessels. In contrast, compliance analysis of the small diameter ELR grafts shows significantly higher elastic performance at all tested pressure pulses than natural human arterial vessels in our study (20/60, 30/70, 40/80, 50/90, 60/100, 80/120, 110/150 mmHg). Vascular grafts are mainly used for the interposition of arterial vessels^7–9,11^, and therefore the comparison of compliance with human arteries is particularly relevant. In cardiovascular tissue engineering, achieving appropriate elasticity is considered a key objective, with most of the cardiovascular implants failing due to high stiffness (low elasticity) and fatigue. On the contrary, our graft has higher initial elasticity than that of the native counterpart. After implantation, in situ remodeling profoundly affects the mechanical behavior of vascular grafts^34^. The deposition of collagen, which provides structural stiffness^35^, typically leads to a natural decrease in compliance over time, bringing the mechanical properties of the graft closer to those of the host vessel. Therefore, a supraphysiological compliance of the graft at the outset is not necessarily detrimental. A graft with slightly higher initial compliance may provide a favorable starting point for achieving physiologically compatible mechanical properties in the long term.

The ELR-microvascular graft must must withstand blood pressure and tensile forces while maintaining sufficient mechanical strength to ensure safe implantation^9^. In the literature, burst pressure or tensile tests are established methods for evaluating the stability of new microvascular techniques before they are tested in living organisms^3,36,37^. Notably, the ELRs microvascular grafts displayed adequate strength of 543 ± 71 mmHg which exceeds approximately 5 times the physiological blood pressure^9^. Historically, it has been proposed that vascular engineering should aim to develop vascular grafts that surpass the properties of natural vessels^38^. However, previous studies have reported sufficient stability of microvascular vessels and microvascular techniques at non-physiological pressures exceeding 300 mmHg^3,36^, which were clearly surpassed by the ELRs biohybrid microvascular grafts in our investigations.

The ELR microvascular graft showed good maneuverability, compatible with a problem-free microsurgery anastomosis procedure. Specifically, the ELR microvascular graft was successfully sutured as vascular interponate between two arteries (AGrA samples), and compared to the current benchmark (i.e. an vein connecting two arteries (AVeA)). Quantitative evaluation of the anastomotic strength revealed that AVeA samples withstand forces of 4.2 ± 1.15 N while AveA counterparts endure values of 3.20 ± 0.65 N. Notably, the AVeA samples failed due to vein rupture, whereas in the AGrA samples, failure occurred either through suture material breakage or arterial rupture. Therefore, the determined strength is higher than the current benchmark used in the clinics for pedicle extension. This indicates that the biohybrid microvascular graft exhibits excellent resistance at the anastomosis site, even under substantial tensile forces.

Previous textile-reinforced ELR concepts described in the literature were developed as larger alloplastic vascular grafts^9,39^, venous valves^19^, and heart valves^25^. In the context of graft development, miniaturization to the small dimensions of just 1 mm diameter imposes a significant challenge, because the smaller you aim that you aim, the more difficult it becomes to reliably produce fine features. Variations that are negligible at larger scales can become critical, impacting graft function and reliability. Here, we have successfully downscaled the fabrication approach to successfully develop such miniaturized grafts for its intended used in microsurgery procedures.

Current vascular models made of silicone effectively mimic haemodynamic conditions and support the development of therapeutic strategies^40^. The quality of these models depends on how faithfully the physiological conditions of the target tissue can be reproduced^16^. However, a critical limitation remains due to the significant discrepancy between the mechanical properties of these model materials and those of native human vascular tissue^40,41^. Since this discrepancy can lead to considerable inaccuracies and uncertainties in the evaluation of novel medical products and surgical techniques due to model materials^41^, the literature frequently describes the validation of microvascular techniques using vessels of animal origin^3,36^ that better mimic the mechanical behaviour of human vessels. To ensure that the evaluation of the novel biohybrid vascular prostheses made of ELRs is as realistic as possible and of the highest quality, the authors decided against the use of model materials or vessels of animal origin and favoured the use of human vessels.

An important limitation of the present study is that the donor vessels used for comparison were obtained from elderly individuals. Although these samples met al.l predefined exclusion criteria, their age-related structural alterations may not accurately represent vascular compliance across a broader population. Vessels such as the radial artery of the upper extremities, which were used in this study, are very rarely subject to vascular wall changes, for example due to arteriosclerosis^42^. It is quite unlikely, but it cannot be ruled out that the observed differences in compliance between native vessels and ELR grafts are partly due to age-related changes in the donor tissue and not to intrinsic properties of the grafts themselves. Future studies including a wider age spectrum of donor vessels would be necessary to more definitively assess physiological compliance benchmarks. However, we recognize that obtaining such tissue presents ethical, practical, and logistical challenges. Nonetheless, the potential influence of donor age on the present findings should be acknowledged when interpreting the compliance data.

## Conclusion

In this ex vivo study, we have successfully developed biohybrid microvascular grafts made of textile-reinforced ELRs that are able to withstand the surgical, technical and functional challenges of a microsurgical demanding procedure, tested here with human vessels. The developed microvascular vessel graft showed higher elastic properties in the evaluation of compliance compared to natural human radial arteries. In addition, they withstood unphysiologically high pressures and showed significantly higher capacity to withstand pulling forces than the gold standard. These grafts have the potential to provide a solution to the pressing need of microvascular interpositions in reconstructive maxillofacial surgery, especially considering that the gold standard (venous autologous vascular interposition graft) is linked to multiple complications. These promising findings of the novel ELRs biohybrid microvascular grafts must be further validated in an animal model in the future.

## Supplementary Information

Below is the link to the electronic supplementary material.

Supplementary Material 1

## Data Availability

The datasets used and/or analyzed during the current study are available from the corresponding author on reasonable request.
