# Supplementary material for: Biohybrid microvascular interponates with integrated elastin-like recombinamers – validation of stability and biomimetic elasticity in human vessels
Source: Sci Rep. 2025 Dec 25;15:44632. doi: 10.1038/s41598-025-33635-x (PMC12749630; doi:10.1038/s41598-025-33635-x)
Supplement: Supplementary file 1 — Supplementary Material 1 [file 41598_2025_33635_MOESM1_ESM.docx]

**Supplementary information**

|  | Amino acid sequence | Mw [Da] |
| --- | --- | --- |
| VKV-ELR | MESLLP VG VPGVG [VPGKG(VPGVG)_5_]_23_  VPGKG VPGVG VPGVG VPGVG VPGV | 60450.9 |

**S 1.** Amino acid sequence of the matrix material (ELR) of the alloplastic vascular grafts


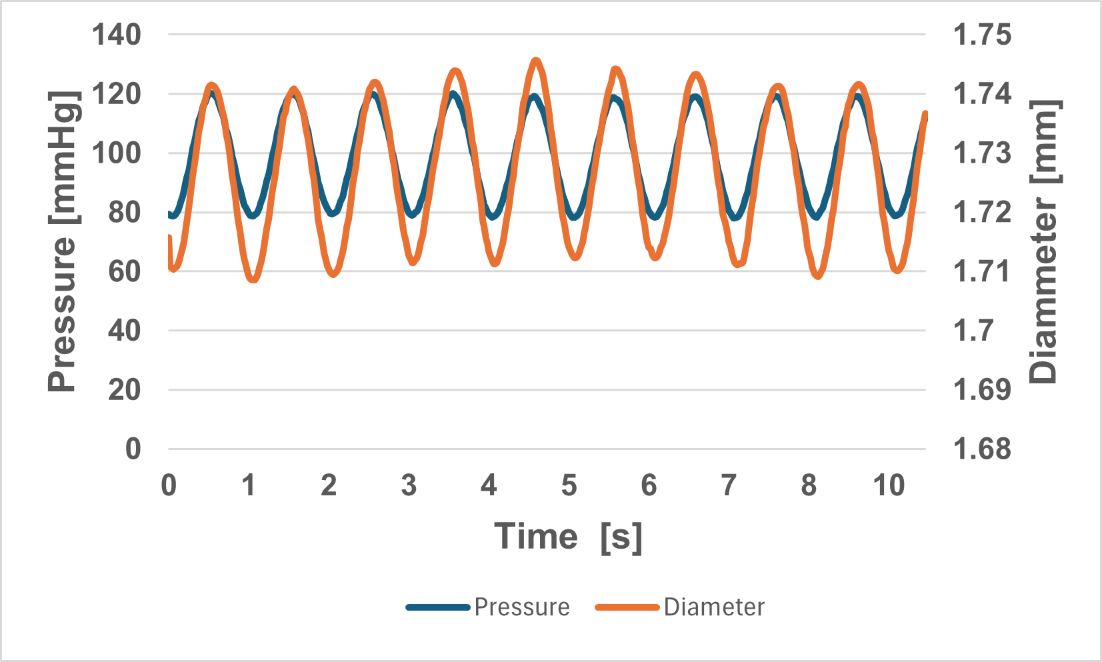


**S 2.**  The graph shows the diameter in relation to pressure over time. The values P1 and P2 correspond to the highest and lowest pressures in each cycle, and D1 and D2 represent the corresponding diameters oft he formula of the vessel compliance test.
